# Supplementary material for: Trends, patterns, determinants and socio-economic inequality of high-risk fertility behavior (HRFB) among Bangladeshi women: Evidence from Bangladesh Demographic and Health Surveys
Source: PLoS One. 2026 Sep 3;21(9):e0356299. doi: 10.1371/journal.pone.0356299 (PMC13541186; doi:10.1371/journal.pone.0356299)
Supplement: Supplementary file 1 [file pone.0356299.s003.pdf]

Reviewer Comments

Summary

The manuscript addresses an important public health issue using nationally representative data from the Bangladesh Demographic and Health Survey. The analysis is generally appropriate, and the findings are potentially valuable. However, a few aspects of clarity and methodological description require minor revision.

Minor Comments

1. **Clarity of Methods in Abstract:** The abstract could be improved for clarity by presenting the methods more clearly. In particular, the definition of high-risk fertility behaviour (HRFB) and the description of the outcome variables are somewhat difficult to follow. Consider revising to achieve a more concise, structured presentation.

2. **Outcome Variables:** The manuscript states that both binary and categorical HRFB variables were constructed, but the rationale is not given. Please briefly justify this choice (e.g., to capture both the occurrence and the intensity of risk) and clearly describe the categorical specification.

3. **Wording Issue:** The phrase "designed on data from" is awkward and should be revised (e.g., "based on data from" or "using data from").

4. **Socioeconomic Disparities:** The sentence describing the assessment of socioeconomic disparities could be streamlined for clarity. Consider revising to:
   "Socioeconomic inequalities in HRFB were assessed using concentration curves and concentration indices (CI)."

5. **Survey Design Specification & Models Goodness-of-fit:** The manuscript would benefit from clearer reporting of the statistical methodology. Specifically, stating that sampling weights were applied is not sufficient for a valid analysis of data from the Bangladesh Demographic and Health Survey. Please clarify whether clustering (PSU) and stratification were also accounted for in the analysis. In addition, no model fit statistics or diagnostic checks (e.g., ICC, AIC/BIC, multicollinearity) are reported. Including these would strengthen the validity and transparency of the analysis.

6. **Wald Confidence Intervals vs. p-values:** Some Wald 95% confidence intervals do not include the null value (0) even though the corresponding p-values are greater than 0.05. Please check the calculation method for the CIs and p-values and clarify which approach was used, particularly for the multilevel logistic and multinomial regression models.

7. **Clarification of Models in Table 2:** Table 2 shows three columns (Any HRFB, Single HRFB, Multiple HRFB), which could be misinterpreted as three separate models. Please clarify whether a single multinomial logistic regression was fitted for the categorical HRFB outcome, with coefficients reported for each comparison, or whether separate models were used.
