# Supplementary material for: Trends, patterns, determinants and socio-economic inequality of high-risk fertility behavior (HRFB) among Bangladeshi women: Evidence from Bangladesh Demographic and Health Surveys
Source: PLoS One. 2026 Sep 3;21(9):e0356299. doi: 10.1371/journal.pone.0356299 (PMC13541186; doi:10.1371/journal.pone.0356299)
Supplement: Supplementary file 2 [file pone.0356299.s004.pdf]

Date: April 04, 2026

Dear Editor,

Thank you for sharing the reviewers' comments for our manuscript entitled "Trends, patterns, determinants and socio-economic inequality of high-risk fertility behavior (HRFB) among Bangladeshi women: evidence from Bangladesh Demographic and Health Survey". We appreciate the time and effort invested by you and the reviewers in providing detailed and constructive feedback.

In response, we have carefully revised the manuscript and addressed all comments point-by-point in the separate file titled "Response to Reviewers." In this document, reviewers' comments are presented in plain text, followed by our responses and a description of the corresponding changes made in the manuscript. A revised version of the manuscript with track changes ("Revised Manuscript with Track Changes") and a clean version ("Manuscript") have also been submitted as requested.
Substantively, we have:

- Added a conceptual framework (now Figure 1) to clarify the theoretical basis for variable selection and the multilevel structure of determinants of HRFB.
- Clarified the specification and justification of the multilevel and multinomial models, including handling of collinearity, model diagnostics, and survey design features.
- Revised sections on temporal trends, COVID-19–related interpretation, and religious affiliation to provide more cautious, nuanced, and non-causal language.
- Corrected errors and inconsistencies in tables, categories, confidence intervals, p-value reporting, and references, and strengthened the description of strengths, limitations, ethics, and data availability.

We have also complied with the journal's technical and policy requirements, including separate submission of figure files, updated ethics and data availability statements, and clarification of consent procedures for the original BDHS 2022 survey.

We hope that the revisions satisfactorily address the concerns raised by the reviewers and that the manuscript is now suitable for publication in PLOS ONE. We are grateful for the opportunity to revise our work and look forward to your further consideration.

Sincerely,
Syed Toukir Ahmed Noor
Corresponding Author

Journal Requirements:

Requirement 1: Please ensure that your manuscript meets PLOS ONE's style requirements, including those for file naming. The PLOS ONE style templates can be found at https://journals.plos.org/plosone/s/file?id=wjVg/PLOSOne_formatting_sample_main_body.pdf and https://journals.plos.org/plosone/s/file?id=ba62/PLOSOne_formatting_sample_title_authors_affiliations.pdf

Response: Thanks for the comment. We reviewed the PLOS ONE style requirements and update the manuscript and file names accordingly, following the provided templates.

Requirement 2: Please provide additional details regarding participant consent. In the ethics statement in the Methods and online submission information, please ensure that you have specified (1) whether consent was informed and (2) what type you obtained (for instance, written or verbal, and if verbal, how it was documented and witnessed). If your study included minors, state whether you obtained consent from parents or guardians. If the need for consent was waived by the ethics committee, please include this information.

If you are reporting a retrospective study of medical records or archived samples, please ensure that you have discussed whether all data were fully anonymized before you accessed them and/or whether the IRB or ethics committee waived the requirement for informed consent. If patients provided informed written consent to have data from their medical records used in research, please include this information.

Response: Thank you for this helpful comment. We have revised the Ethics section to clarify that the 2022 BDHS obtained ethical approval from the ICF Institutional Review Board and the Bangladesh Medical Research Council (BMRC), and that informed, voluntary consent was obtained from all participants, with parental/guardian consent for minors. We also state that the publicly available BDHS datasets are fully anonymized, so no additional approval or consent was required for this secondary analysis. These details are now included in the revised manuscript

Requirement 3: In the online submission form, you indicated that data is publicly available on request on the DHS websites (https://www.dhsprogram.com/data/available-datasets.cfm).

All PLOS journals now require all data underlying the findings described in their manuscript to be freely available to other researchers, either a. In a public repository, b. Within the manuscript itself, or c. Uploaded as supplementary information.

This policy applies to all data except where public deposition would breach compliance with the protocol approved by your research ethics board. If your data cannot be made publicly available for ethical or legal reasons (e.g., public availability would compromise patient privacy), please explain your reasons on resubmission and your exemption request will be escalated for approval.

Response: Thank you for this clarification. In addition to noting that the original BDHS data are accessible upon registration through the DHS Program, we have now provided the dataset underlying our analyses as an Excel file in the Supporting Information (S1 File). This ensures that all data used in the manuscript are freely available to other researchers in accordance with the PLOS data availability policy.

Requirement 4: When completing the data availability statement of the submission form, you indicated that you will make your data available on acceptance. We strongly recommend all authors decide on a data sharing plan before acceptance, as the process can be lengthy and hold up publication timelines. Please note that, though access restrictions are acceptable now, your entire data will need to be made freely accessible if your manuscript is accepted for publication. This policy applies to all data except where public deposition would breach compliance with the protocol approved by your research ethics board. If you are unable to adhere to our open data policy, please kindly revise your statement to explain your reasoning and we will seek the editor's input on an exemption. Please be assured that, once you have provided your new statement, the assessment of your exemption will not hold up the peer review process.

Response: Thank you for this important clarification. The primary data used in this study are individual-level microdata from the Bangladesh Demographic and Health Survey (BDHS) 2022, which are owned and managed by the DHS Program. Access to these datasets requires prior registration and

approval from the DHS Program, and our data-use agreement does not permit us to republish or publicly deposit the microdata (or any subset thereof) under an open licence.
In light of this, we are unable to make the full BDHS 2022 dataset freely available in a public repository. We have therefore revised our Data Availability statement to clarify that: (i) all BDHS 2022 datasets can be obtained directly from the DHS Program upon registration and approval (https://www.dhsprogram.com/data/available-datasets.cfm), and (ii) we provide the complete Stata do-file used for data cleaning, variable construction, survey weighting, and all analyses as Supporting Information, so that any researcher who gains access to the BDHS 2022 data can fully reproduce our results.
Because restricting public redistribution of the DHS microdata is a condition of the original data-use protocol and data-ownership policy, we kindly request an exemption from the requirement to deposit the full analysis dataset in an open repository. We hope that the combination of controlled access via the DHS Program and fully shared analysis code will be acceptable under PLOS ONE's data policy.

Requirement 5: Please ensure that you refer to Figure 2 in your text as, if accepted, production will need this reference to link the reader to the figure.
Response: Thank you for this reminder. In the revised manuscript, the former Figure 2 has been renumbered as Figure 3, and it is already explicitly cited in the main text to guide readers to the figure.

Requirement 6: Please remove your figures from within your manuscript file, leaving only the individual TIFF/EPS image files, uploaded separately. These will be automatically included in the reviewers' PDF.
Response: Thank you for the instruction. All figures have been removed from the main manuscript file, and the individual figure files have been uploaded separately in TIFF format as requested.

Requirement 7: We note that Figure 2 in your submission contain map images which may be copyrighted. All PLOS content is published under the Creative Commons Attribution License (CC BY 4.0), which means that the manuscript, images, and Supporting Information files will be freely available online, and any third party is permitted to access, download, copy, distribute, and use these materials in any way, even commercially, with proper attribution. For these reasons, we cannot publish previously copyrighted maps or satellite images created using proprietary data, such as Google software (Google Maps, Street View, and Earth). For more information, see our copyright guidelines: http://journals.plos.org/plosone/s/licenses-and-copyright.
We require you to either (a) present written permission from the copyright holder to publish these figures specifically under the CC BY 4.0 license, or (b) remove the figures from your submission:

a. You may seek permission from the original copyright holder of Figure 2 to publish the content specifically under the CC BY 4.0 license.
We recommend that you contact the original copyright holder with the Content Permission Form (http://journals.plos.org/plosone/s/file?id=7c09/content-permission-form.pdf) and the following text:
"I request permission for the open-access journal PLOS ONE to publish XXX under the Creative Commons Attribution License (CCAL) CC BY 4.0 (http://creativecommons.org/licenses/by/4.0/). Please be aware that this license allows unrestricted use and distribution, even commercially, by third parties. Please reply and provide explicit written permission to publish XXX under a CC BY license and complete the attached form."
Please upload the completed Content Permission Form or other proof of granted permissions as an ""Other"" file with your submission.
In the figure caption of the copyrighted figure, please include the following text: "Reprinted from [ref] under a CC BY license, with permission from [name of publisher], original copyright [original copyright year]."

b. If you are unable to obtain permission from the original copyright holder to publish these figures under the CC BY 4.0 license or if the copyright holder's requirements are incompatible with the CC BY 4.0 license, please either i) remove the figure or ii) supply a replacement figure that complies with the CC BY 4.0 license. Please check copyright information on all replacement figures and update the

figure caption with source information. If applicable, please specify in the figure caption text when a figure is similar but not identical to the original image and is therefore for illustrative purposes only.
The following resources for replacing copyrighted map figures may be helpful:
USGS National Map Viewer (public domain): http://viewer.nationalmap.gov/viewer/
The Gateway to Astronaut Photography of Earth (public domain): http://eol.jsc.nasa.gov/sseop/clickmap/
Maps at the CIA (public domain): https://www.cia.gov/library/publications/the-world-factbook/index.html and https://www.cia.gov/library/publications/cia-maps-publications/index.html
NASA Earth Observatory (public domain): http://earthobservatory.nasa.gov/
Landsat: http://landsat.visibleearth.nasa.gov/
USGS EROS (Earth Resources Observatory and Science (EROS) Center) (public domain): http://eros.usgs.gov/#
Natural Earth (public domain): http://www.naturalearthdata.com/
Response: Thank you for raising this issue. Thank you for your comment regarding Figure 3. We confirm that the map is not subject to copyright restrictions. The figure was created entirely by the authors using ArcGIS version 10.8 to illustrate the division-wise distribution of HRFB across the different divisions of Bangladesh. The divisional shapefile used to prepare this map was obtained from the Humanitarian Data Exchange (HDX), an open-source platform managed by the United Nations Office for the Coordination of Humanitarian Affairs (https://data.humdata.org/dataset/cod-ab-bgd), which is freely available for public use without licensing restrictions. As the map was generated from open-source data and original analysis, it fully complies with the Creative Commons Attribution License (CC BY 4.0) requirements for publication in PLOS ONE.

Requirement 8: If the reviewer comments include a recommendation to cite specific previously published works, please review and evaluate these publications to determine whether they are relevant and should be cited. There is no requirement to cite these works unless the editor has indicated otherwise.
Response: Thank you for this clarification

Response Reviewer's Comments

Reviewer #1:

Comment 1: Material and Methods: Please draw a flowchart to illustrate the conceptual framework.
Response: Thank you for this helpful suggestion. We agree that the earlier version did not sufficiently explain the rationale for selecting explanatory variables. We have now added a brief conceptual framework in the Methods section (Fig 1), outlining the pathways linking sociodemographic, household , and community-level factors to HRFB and citing the key theoretical and empirical literature that informed our covariate selection.

Comment 2: Figures: Map - Add a title
Response: Thank you for these helpful suggestion. We have also added an informative title to the map to improve its clarity and interpretability (Fig 3).

Reviewer #2:

Comment 1: Missing justification for variable selection: While the authors state they "reviewed the most recent relevant articles" (Line 130), they do not provide a theoretical framework or conceptual model guiding variable selection. Add a brief conceptual framework or cite specific theoretical models that informed covariate selection.
Response: Thank you for this helpful suggestion. We agree that the earlier version did not sufficiently explain the rationale for selecting explanatory variables. We have now added a brief conceptual framework in the Methods section (Fig 1), outlining the pathways linking sociodemographic, household , and community-level factors to HRFB and citing the key theoretical and empirical literature that informed our covariate selection.

Comment 2: Husband's age exclusion (Line 156): The authors excluded the husband's age due to high collinearity, but do not report:
• The VIF values that led to this decision.
• Whether sensitivity analyses were conducted with/without this variable.
• The first two categories for husband's age in Table 1 overlap at age 30. A husband who is exactly 30 years old could logically be placed in either category. This violates the statistical principle that categories should be mutually exclusive and collectively exhaustive. If these categories were used in the regression (Table 4), the categorization error would affect the results (though husband's age was excluded from the regression due to collinearity, the descriptive table is still incorrect).
• Potential implications of excluding an important demographic factor.
• Is excluding variables the best solution? Consider adding possible alternative solutions to the limitations (DOI: 10.1080/03610918.2019.1711409).
Response: Thank you for these detailed and helpful comments. We apologize for the confusion caused by the overlapping husband's age categories in Tables 1 and 3. This was a typographical error: the first category should have been 15–29 years, and the second 30–34 years. We have corrected these categories so that they are now mutually exclusive and collectively exhaustive, and a husband who is exactly 30 years old is included in the 30–34 years group in the revised tables. Regarding collinearity, we have now reported the Variance Inflation Factor (VIF) that led to the exclusion of husband's age from the multivariable models. The VIF for husband's age was 6.72, which we considered indicative of problematic multicollinearity. We have added this information to the Statistical Analysis subsection of the Methods (line 168-173). We also conducted sensitivity analyses including and excluding husband's age from the multivariable models. These analyses showed that the direction and magnitude of the associations between the main covariates and HRFB were materially unchanged when husband's age was included. We agree that excluding an important demographic factor is not ideal. In the Limitations section (line 407-411), we have added a short paragraph noting that we opted to exclude husband's age due to multicollinearity, and we acknowledge that alternative approaches to handling multicollinearity (as discussed in the referenced article) could be considered in future work.

Comment 3: Causal attribution to COVID-19 (Lines 284-291): The authors attribute the slight increase in HRFB from 2017-18 (57%) to 2022 (60%) to the COVID-19 pandemic. Soften this claim or present it as a hypothesis requiring further investigation. That is, the causal claim is speculative without:
• Supporting evidence or citations demonstrating pandemic impacts on HRFB specifically.
• Consideration of other potential explanations (e.g., sampling variation, changing demographics).
Response: We thank the reviewer for this important comment. We agree that our initial wording could be interpreted as implying a causal effect of the COVID-19 pandemic on the observed increase in HRFB. We have therefore revised the discussion to present the pandemic as a possible contributing factor rather than a definitive cause and to acknowledge that this interpretation is speculative and would require further investigation with appropriate data. We also note that other explanations, such as sampling variation and underlying demographic or service-related changes, may have contributed to the observed difference. (line 310-317)

Comment 4: Religious affiliation interpretation (Lines 331-343): The finding that Muslim women have higher odds of multiple HRFB is presented with potentially overgeneralized explanations about "misconceptions about Islamic teachings" and "cultural norms." This requires:
• More nuanced discussion acknowledging diversity within religious groups.
• Consideration of confounding factors not fully captured in models.
• Careful language to avoid stereotyping or oversimplification.
• Revision to emphasize that religious affiliation may be a proxy for other unmeasured factors (e.g., community norms, healthcare access) rather than religion itself being causative.
Response: We thank the reviewer for this important and thoughtful comment. We fully agree that the association between religious affiliation and multiple HRFB requires nuanced interpretation and should not be framed in a way that risks stereotyping or implying causality.
In response, we have substantially revised the relevant paragraph in the Discussion (now Lines 361–368). First, we have clarified that religious affiliation is unlikely to be causative in itself and may instead act as a proxy for unmeasured contextual factors such as community norms, gender relations, and structural barriers to healthcare. Second, we have removed wording that could be interpreted as overgeneralizing (e.g. 'misconceptions about Islamic teachings') and now explicitly acknowledge the heterogeneity within Muslim populations and the diversity of interpretations and practices across settings. Third, we now highlight that our models may not fully capture all confounding factors (including local service availability, quality of counseling, and neighborhood-level norms), and we encourage future research to explore these mechanisms in more depth. We believe these changes result in a more cautious and respectful interpretation of the observed association.

Comment 5: In Table 2, some CIs are asymmetrical (e.g., "16.45(16.49,17.36)" appears to have an error as the lower bound exceeds the point estimate). Standardize reporting to 2 decimal places throughout and verify all intervals.
Response: Thank you for pointing this out. We have carefully reviewed **Table 2,** corrected the errors in the confidence intervals, and standardized the reporting of estimates and CIs to two decimal places throughout the table.

Comment 6: Some p-values are reported as "p<0.01" while others use asterisks. Consider using a consistent system (either all p-values or all asterisks).
Response: Thank you for the helpful comment. We understand the preference for a single format; however, we have retained exact p-values in the text and Table 3 because they provide more precise information, while using asterisks only in Table 4, where space is limited. To avoid confusion, we have also added a note explaining the notation used.

Comment 7: Multilevel model specification: The authors do not clearly specify:
• Whether random intercepts or random slopes were used.
• The levels of clustering (household? community? district?)
• Justification of the multilevel approach.
• The authors did not use interaction terms in their models, and this is a limitation that should be

addressed.

• The manuscript mentions only the Variance Inflation Factor (VIF) for multicollinearity assessment, but is completely silent on other essential model diagnostics.

Response: Thank you for these important methodological comments. We have now clarified the specification and justification of our multilevel models. Following DHS guidelines, we applied sampling weights and accounted for the complex survey design, incorporating primary sampling units (PSUs) and stratification to ensure national representativeness. Given the hierarchical structure of the BDHS data, with individuals nested within households and households nested within communities, we fitted multilevel mixed-effects logistic regression models with random intercepts at the cluster (PSU) level. This allows the baseline odds of HRFB to vary across clusters while assuming fixed slopes for the covariates.

We have added a brief justification for the use of multilevel modelling in the Methods section and specified that the random effects were defined at the cluster level. We also acknowledge that we did not include interaction terms in the current models and now note this explicitly as a limitation, indicating that future work should explore potential effect modification (e.g. by wealth or place of residence). In addition to reporting VIF for multicollinearity, we now describe further model diagnostics, including intraclass correlation coefficients (ICCs) and model fit statistics (e.g. AIC/BIC), and we summarize these in the Results. (line 165-171)

Comment 8: Missing data handling: The manuscript does not describe how missing data were handled (DOI: 10.19139/soic-2310-5070-2894). Given the large sample, even small percentages missing could affect the results.

Response: Thank you for this important suggestion. We have added a description of the procedure for handling missing data in the Methods section. (line 171-173)

Comment 9: Include a brief subsection in the Discussion explicitly linking findings to current Bangladesh health policies.

Response: Thank you for this important suggestion. We have added a brief subsection in the Discussion explicitly linking findings to current Bangladesh health policies (lines 383-390).

Comment 10: Reproducibility: Provide the exact code used for data cleaning, variable creation (e.g., the HRFB categories), weighting, and all models (multilevel logistic and multinomial, concentration indices). The authors should add a line in the Data Availability, Methods, or Supporting Information section of the manuscript: "The Stata do-file used for the data analysis is available as Supporting Information (S1 File)."

Response: Thank you for emphasizing this important aspect. We have prepared the complete Stata do-file used for data cleaning, variable construction (including the HRFB categories), application of survey weights, and all statistical analyses (multilevel logistic and multinomial models, as well as concentration indices). As recommended, we have added the following statement to the Data Availability/Supporting Information section of the manuscript: 'The Stata do-file used for the data analysis is available as Supporting Information (S1 File).' This will enable full reproducibility of our analyses.

Comment 11: The authors need to demonstrate that their use of the chi-squared test was appropriate, given their data structure, and that the key assumptions were met.

Response: Thank you for this important comment. We have clarified in the Methods section that the chi-squared test was used only for categorical variables to assess bivariate associations. The observations were independent, and the expected cell counts met the usual assumptions for the test, with no substantial violation of minimum expected frequency requirements. Therefore, the chi-squared test was appropriate for our data structure (line 157-159).

Comment 12: The totals in Table 1 are inconsistent across variables, which suggests either:

• Missing data not properly accounted for.

• Reporting errors.

• Major subsample without explanation.
Recommendation: The authors must revise Table 1 to clearly indicate the base population for each variable and ensure all frequencies within each subsample are consistent. This is essential for transparency and reproducibility.
Response: Thank you for carefully examining Table 1. We have revised Table 1 to ensure that the totals for each variable are internally consistent and clearly indicate the base population. This should improve transparency and reproducibility.

Comment 13: Using “1” in Table 4 to indicate reference categories could be misinterpreted. I strongly recommend changing all “1” entries to "Ref." and adding a clear footnote.
Response: Thank you for this suggestion. We have changed all the “1” entries to “Ref” and added a footnote for understanding.

Comment 14: References are inconsistently formatted (some with DOIs, some without; some with truncated). PLOS ONE has specific reference formatting requirements that should be followed.
• In the text (Lines 171-172): The authors write: “The CIX was calculated using the 'convenient covariance' formula proposed by O'Donnell et al. [41].” In the reference list: [41] is Jann B. (2016). This is a miscitation. The authors must fix this citation error and re-verify all subsequent reference numbers.
• [27] and [56] are the same.
• [29] and [33] are the same.
• [31] and [62] are the same.
• There is no reference [63] in the list.
• The authors should carefully review their entire reference list and all in-text citations to ensure consistency and accuracy.
Response: Thank you for highlighting these issues. We have carefully reviewed the entire reference list and all in-text citations to ensure consistency and accuracy in line with PLOS ONE guidelines. Specifically, we corrected the miscitation of the convenient covariance formula, resolved duplicated references, addressed the missing and other formatting discrepancies.

Comment 15: The paper contains enough typos and grammatical errors to require a thorough language revision.
Response: Thank you for this comment. The manuscript has undergone a thorough language revision, and typographical and grammatical errors have been corrected to improve clarity and readability.

Reviewer #3:

Comment 1: Clarity of Methods in Abstract: The abstract could be improved for clarity by presenting the methods more clearly. In particular, the definition of high-risk fertility behaviour (HRFB) and the description of the outcome variables are somewhat difficult to follow. Consider revising to achieve a more concise, structured presentation.
Response: Thank you for this useful suggestion. We have revised the Methods section of the abstract to provide a clearer and more concise description of the definition of HRFB and the outcome variables, using a more structured presentation (Lines 26–28).

Comment 2: Outcome Variables: The manuscript states that both binary and categorical HRFB variables were constructed, but the rationale is not given. Please briefly justify this choice (e.g., to capture both the occurrence and the intensity of risk) and clearly describe the categorical specification.
Response: Thank you for this important comment. We have added a brief explanation in the Methods section (Lines 126–131) to clarify that we constructed both a binary “HRFB status” variable (any vs. none) to capture the overall occurrence and a categorical variable “HRFB level” (no, single, multiple HRFB) to capture the intensity and pattern of risk. We also describe the categorical specification more explicitly.

Comment 3: Wording Issue: The phrase ``designed on data from'' is awkward and should be revised (e.g., ``based on data from'' or ``using data from'').
Response: Thank you for this suggestion. We have revised the phrase in the manuscript.

Comment 4: Socioeconomic Disparities: The sentence describing the assessment of socioeconomic disparities could be streamlined for clarity. Consider revising to: ``Socioeconomic inequalities in HRFB were assessed using concentration curves and concentration indices (CIX).''
Response: Thank you for this helpful wording suggestion. We have revised the sentence to: "Socioeconomic inequalities in HRFB were assessed using concentration curves and concentration indices (CIX).".

Comment 5: Survey Design Specification and Models Goodness-of-fit: The manuscript would benefit from clearer reporting of the statistical methodology. Specifically, stating that sampling weights were applied is not sufficient for a valid analysis of data from the {Bangladesh Demographic and Health Survey}. Please clarify whether clustering (PSU) and stratification were also accounted for in the analysis. In addition, no model fit statistics or diagnostic checks (e.g., ICC, AIC/BIC, multicollinearity) are reported. Including these would strengthen the validity and transparency of the analysis.
Response: Thank you for these important methodological points. We now clarify that our analyses accounted for the full BDHS survey design, including sampling weights, clustering (PSU), and stratification (Lines 145–173). In addition, we report key model fit statistics and diagnostics (ICC, AIC and BIC) in the Results

Comment 6: Wald Confidence Intervals vs. p-values: Some Wald 95\% confidence intervals do not include the null value (0) even though the corresponding p-values are greater than 0.05. Please check the calculation method for the CIs and p-values and clarify which approach was used, particularly for the multilevel logistic and multinomial regression models.
Response: Thank you for this important observation. We have rechecked all reported confidence intervals and p-values in the regression tables. The estimates in these models are adjusted odds ratios (AORs) and relative risk ratios (RRRs), so the appropriate null value for the 95% confidence intervals is 1 rather than 0. Accordingly, intervals that do not include 1 correspond to p-values <0.05, and intervals that include 1 correspond to p-values ≥ 0.05. We have ensured that the CIs and p-values are now consistent throughout the multilevel logistic and multinomial regression results.

Comment 7: Clarification of Models in Table 2: Table 2 shows three columns (Any HRFB, Single HRFB, Multiple HRFB), which could be misinterpreted as three separate models. Please clarify whether a single multinomial logistic regression was fitted for the categorical HRFB outcome, with coefficients reported for each comparison, or whether separate models were used.
Response: Thank you for this helpful comment. We apologize for the lack of clarity. Table 2 does not present results from regression models. Instead, it shows the bivariate distributions of any HRFB, single HRFB, and multiple HRFB across the explanatory variables, based on cross-tabulations and chi-squared tests. No multinomial or other regression model was used for this table.
