# Supplementary material for: Trends, patterns, determinants and socio-economic inequality of high-risk fertility behavior (HRFB) among Bangladeshi women: Evidence from Bangladesh Demographic and Health Surveys
Source: PLoS One. 2026 Sep 3;21(9):e0356299. doi: 10.1371/journal.pone.0356299 (PMC13541186; doi:10.1371/journal.pone.0356299)
Supplement: Supplementary file 3 [file pone.0356299.s002.docx]

**Table S1.** Comparison of adjusted odds ratios for any high-risk fertility behaviour (HRFB) from the primary multilevel logistic regression model and a sensitivity model including husband's age.

| **Variable** | **Model 1 (no husband's age) AOR (95% CI)** | **Sensitivity Model (husband's age included) AOR (95% CI)** |
| --- | --- | --- |
| **Age at marriage** |  |  |
| <18 | Ref | Ref |
| ≥18 | 0.91 (0.85–0.98)* | 0.91 (0.85–0.98)* |
| **Respondent's education** |  |  |
| No education | Ref | Ref |
| Primary | 0.54 (0.48–0.61)*** | 0.53 (0.47–0.60)*** |
| Secondary | 0.29 (0.25–0.32)*** | 0.28 (0.25–0.32)*** |
| Higher | 0.18 (0.15–0.20)*** | 0.17 (0.14–0.20)*** |
| **Religion** |  |  |
| Non-Muslim | Ref | Ref |
| Muslim | 0.96 (0.87–1.07) | 0.96 (0.87–1.07) |
| **Media exposure** |  |  |
| No | Ref | Ref |
| Yes | 0.86 (0.80–0.92)*** | 0.86 (0.80–0.92)*** |
| **Currently using contraceptive method** |  |  |
| No | Ref | Ref |
| Yes | 1.05 (0.98–1.11) | 1.05 (0.98–1.12) |
| **Husband's education** |  |  |
| No education | Ref | Ref |
| Primary | 0.69 (0.63–0.76)*** | 0.69 (0.62–0.76)*** |
| Secondary | 0.62 (0.56–0.69)*** | 0.61 (0.55–0.68)*** |
| Higher | 0.82 (0.72–0.93)** | 0.80 (0.71–0.91)*** |
| **Husband's age** |  |  |
| <30 | — | Ref |
| 30–34 | — | 0.91 (0.82–1.02) |
| 35–39 | — | 1.54 (1.39–1.70)*** |
| 40–44 | — | 4.20 (3.76–4.69)*** |
| 45–49 | — | 9.21 (8.07–10.51)*** |
| 50+ | — | 15.25 (13.39–17.37)*** |
| **Number of household members** |  |  |
| ≤4 | Ref | Ref |
| 5 or more | 1.27 (1.19–1.35)*** | 1.27 (1.19–1.35)*** |
| **Wealth index** |  |  |
| Richest | Ref | Ref |
| Fourth | 0.91 (0.84–0.97)** | 0.99 (0.89–1.10 |
| Middle | 1.02 (0.95–1.10) | 1.13 (0.99–1.27 |
| Second | 1.06 (0.98–1.14) | 1.06 (0.93–1.20 |
| Poorest | 1.22 (1.13–1.32)*** | 1.17 (1.02–1.35)*** |
| **Place of residence** |  |  |
| Urban | Ref | Ref |
| Rural | 1.08 (1.01–1.16)* | 1.09 (1.00–1.18)* |
| **Division** |  |  |
| Dhaka | Ref | Ref |
| Barishal | 1.28 (1.13–1.44)*** | 1.29 (1.12–1.48)*** |
| Chattogram | 1.15 (1.03–1.28)* | 1.15 (1.01–1.31)* |
| Khulna | 1.02 (0.91–1.15) | 1.03 (0.90–1.17) |
| Mymensingh | 1.34 (1.18–1.52)*** | 1.35 (1.17–1.56)*** |
| Rajshahi | 1.06 (0.94–1.18) | 1.06 (0.93–1.20) |
| Rangpur | 1.12 (1.00–1.27) | 1.13 (0.99–1.30) |
| Sylhet | 1.04 (0.92–1.18) | 1.04 (0.90–1.20) |

*p<0.05, **p<0.01, ***p<0.001
