# Supplementary material for: Trends, patterns, determinants and socio-economic inequality of high-risk fertility behavior (HRFB) among Bangladeshi women: Evidence from Bangladesh Demographic and Health Surveys
Source: PLoS One. 2026 Sep 3;21(9):e0356299. doi: 10.1371/journal.pone.0356299 (PMC13541186; doi:10.1371/journal.pone.0356299)
Supplement: Peer review history

# Peer review: Decision Letter 0

**DOI:** 10.1371/journal.pone.0356299.r001

**Authors:** Ahmed Jubayer

25 Mar 2026

Dear Dr. Noor,

Thank you for submitting your manuscript to PLOS ONE. After careful consideration, we feel that it has merit but does not fully meet PLOS ONE’s publication criteria as it currently stands. Therefore, we invite you to submit a revised version of the manuscript that addresses the points raised during the review process.

Please submit your revised manuscript by May 09 2026 11:59PM. If you will need more time than this to complete your revisions, please reply to this message or contact the journal office at plosone@plos.org. When you’re ready to submit your revision, log on to https://www.editorialmanager.com/pone/ and select the 'Submissions Needing Revision' folder to locate your manuscript file.

- A letter that responds to each point raised by the academic editor and reviewer(s). You should upload this letter as a separate file labeled 'Response to Reviewers'.
- A marked-up copy of your manuscript that highlights changes made to the original version. You should upload this as a separate file labeled 'Revised Manuscript with Track Changes'.
- An unmarked version of your revised paper without tracked changes. You should upload this as a separate file labeled 'Manuscript'.

If you would like to make changes to your financial disclosure, please include your updated statement in your cover letter. Guidelines for resubmitting your figure files are available below the reviewer comments at the end of this letter.

If applicable, we recommend that you deposit your laboratory protocols in protocols.io to enhance the reproducibility of your results. Protocols.io assigns your protocol its own identifier (DOI) so that it can be cited independently in the future. For instructions see: https://journals.plos.org/plosone/s/submission-guidelines#loc-laboratory-protocols. Additionally, PLOS ONE offers an option for publishing peer-reviewed Lab Protocol articles, which describe protocols hosted on protocols.io. Read more information on sharing protocols at https://plos.org/protocols?utm_medium=editorial-email&utm_source=authorletters&utm_campaign=protocols.

As the corresponding author, your ORCID iD is verified in the submission system and will appear in the published article. PLOS supports the use of ORCID, and we encourage all coauthors to register for an ORCID iD and use it as well. Please encourage your coauthors to verify their ORCID iD within the submission system before final acceptance, as unverified ORCID iDs will not appear in the published article. *Only*  the individual author can complete the verification step; PLOS staff *cannot*  verify ORCID iDs on behalf of authors.

We look forward to receiving your revised manuscript.

Kind regards,

Ahmed Jubayer

Academic Editor

PLOS One

Journal Requirements:

When submitting your revision, we need you to address these additional requirements.

1. Please ensure that your manuscript meets PLOS ONE's style requirements, including those for file naming. The PLOS ONE style templates can be found at

https://journals.plos.org/plosone/s/file?id=wjVg/PLOSOne_formatting_sample_main_body.pdf and

https://journals.plos.org/plosone/s/file?id=ba62/PLOSOne_formatting_sample_title_authors_affiliations.pdf

2. Please provide additional details regarding participant consent. In the ethics statement in the Methods and online submission information, please ensure that you have specified (1) whether consent was informed and (2) what type you obtained (for instance, written or verbal, and if verbal, how it was documented and witnessed). If your study included minors, state whether you obtained consent from parents or guardians. If the need for consent was waived by the ethics committee, please include this information.

If you are reporting a retrospective study of medical records or archived samples, please ensure that you have discussed whether all data were fully anonymized before you accessed them and/or whether the IRB or ethics committee waived the requirement for informed consent. If patients provided informed written consent to have data from their medical records used in research, please include this information.

3. In the online submission form, you indicated that data is publicly available on request on the DHS websites (https://www.dhsprogram.com/data/available-datasets.cfm).

All PLOS journals now require all data underlying the findings described in their manuscript to be freely available to other researchers, either a. In a public repository, b. Within the manuscript itself, or c. Uploaded as supplementary information.

This policy applies to all data except where public deposition would breach compliance with the protocol approved by your research ethics board. If your data cannot be made publicly available for ethical or legal reasons (e.g., public availability would compromise patient privacy), please explain your reasons on resubmission and your exemption request will be escalated for approval.

4. When completing the data availability statement of the submission form, you indicated that you will make your data available on acceptance. We strongly recommend all authors decide on a data sharing plan before acceptance, as the process can be lengthy and hold up publication timelines. Please note that, though access restrictions are acceptable now, your entire data will need to be made freely accessible if your manuscript is accepted for publication. This policy applies to all data except where public deposition would breach compliance with the protocol approved by your research ethics board. If you are unable to adhere to our open data policy, please kindly revise your statement to explain your reasoning and we will seek the editor's input on an exemption. Please be assured that, once you have provided your new statement, the assessment of your exemption will not hold up the peer review process.

5. Please ensure that you refer to Figure 2 in your text as, if accepted, production will need this reference to link the reader to the figure.

6. Please remove your figures from within your manuscript file, leaving only the individual TIFF/EPS image files, uploaded separately. These will be automatically included in the reviewers’ PDF.

7. We note that Figure 2 in your submission contain map images which may be copyrighted. All PLOS content is published under the Creative Commons Attribution License (CC BY 4.0), which means that the manuscript, images, and Supporting Information files will be freely available online, and any third party is permitted to access, download, copy, distribute, and use these materials in any way, even commercially, with proper attribution. For these reasons, we cannot publish previously copyrighted maps or satellite images created using proprietary data, such as Google software (Google Maps, Street View, and Earth). For more information, see our copyright guidelines: http://journals.plos.org/plosone/s/licenses-and-copyright.

We require you to either (a) present written permission from the copyright holder to publish these figures specifically under the CC BY 4.0 license, or (b) remove the figures from your submission:

a. You may seek permission from the original copyright holder of Figure 2 to publish the content specifically under the CC BY 4.0 license.

We recommend that you contact the original copyright holder with the Content Permission Form (http://journals.plos.org/plosone/s/file?id=7c09/content-permission-form.pdf) and the following text:

“I request permission for the open-access journal PLOS ONE to publish XXX under the Creative Commons Attribution License (CCAL) CC BY 4.0 (http://creativecommons.org/licenses/by/4.0/). Please be aware that this license allows unrestricted use and distribution, even commercially, by third parties. Please reply and provide explicit written permission to publish XXX under a CC BY license and complete the attached form.”

Please upload the completed Content Permission Form or other proof of granted permissions as an ""Other"" file with your submission.

In the figure caption of the copyrighted figure, please include the following text: “Reprinted from [ref] under a CC BY license, with permission from [name of publisher], original copyright [original copyright year].”

b. If you are unable to obtain permission from the original copyright holder to publish these figures under the CC BY 4.0 license or if the copyright holder’s requirements are incompatible with the CC BY 4.0 license, please either i) remove the figure or ii) supply a replacement figure that complies with the CC BY 4.0 license. Please check copyright information on all replacement figures and update the figure caption with source information. If applicable, please specify in the figure caption text when a figure is similar but not identical to the original image and is therefore for illustrative purposes only.

The following resources for replacing copyrighted map figures may be helpful:

USGS National Map Viewer (public domain): http://viewer.nationalmap.gov/viewer/

The Gateway to Astronaut Photography of Earth (public domain): http://eol.jsc.nasa.gov/sseop/clickmap/

Maps at the CIA (public domain): https://www.cia.gov/library/publications/the-world-factbook/index.html and https://www.cia.gov/library/publications/cia-maps-publications/index.html

NASA Earth Observatory (public domain): http://earthobservatory.nasa.gov/

Landsat: http://landsat.visibleearth.nasa.gov/

USGS EROS (Earth Resources Observatory and Science (EROS) Center) (public domain): http://eros.usgs.gov/#

Natural Earth (public domain): http://www.naturalearthdata.com/

8. If the reviewer comments include a recommendation to cite specific previously published works, please review and evaluate these publications to determine whether they are relevant and should be cited. There is no requirement to cite these works unless the editor has indicated otherwise.

[Note: HTML markup is below. Please do not edit.]

Reviewers' comments:

Reviewer's Responses to Questions

**Comments to the Author**

1. Is the manuscript technically sound, and do the data support the conclusions?

Reviewer #1: Yes

Reviewer #2: Partly

Reviewer #3: Yes

**********

2. Has the statistical analysis been performed appropriately and rigorously?

Reviewer #1: Yes

Reviewer #2: No

Reviewer #3: No

**********

3. Have the authors made all data underlying the findings in their manuscript fully available?

The PLOS Data policy

Reviewer #1: No

Reviewer #2: Yes

Reviewer #3: Yes

**********

4. Is the manuscript presented in an intelligible fashion and written in standard English?

Reviewer #1: Yes

Reviewer #2: No

Reviewer #3: Yes

**********

Reviewer #1: The study is highly relevant and addresses a critical issue. However, few revisions are recommended:

Material and Methods

Please draw a flowchart to illustrate the conceptual framework.

Figures

Map - Add a title

Reviewer #2: Journal: PLOS ONE

MS ID: PONE-D-25-25913

Title: Trends, patterns, determinants and socio-economic inequality of high-risk fertility behavior (HRFB) among Bangladeshi women: evidence from Bangladesh Demographic Health Survey 2022

Version: 1 Date: 28 February 2026

Reviewer's report:

Comment 1—Missing justification for variable selection: While the authors state they “reviewed the most recent relevant articles” (Line 130), they do not provide a theoretical framework or conceptual model guiding variable selection. Add a brief conceptual framework or cite specific theoretical models that informed covariate selection.

Comment 2—Husband's age exclusion (Line 156): The authors excluded the husband's age due to high collinearity, but do not report:

• The VIF values that led to this decision.

• Whether sensitivity analyses were conducted with/without this variable.

• The first two categories for husband's age in Table 1 overlap at age 30. A husband who is exactly 30 years old could logically be placed in either category. This violates the statistical principle that categories should be mutually exclusive and collectively exhaustive. If these categories were used in the regression (Table 4), the categorization error would affect the results (though husband's age was excluded from regression due to collinearity, the descriptive table is still incorrect).

• Potential implications of excluding an important demographic factor.

• Is excluding variables the best solution? Consider adding possible alternative solutions to the limitations (DOI: 10.1080/03610918.2019.1711409).

Comment 3—Causal attribution to COVID-19 (Lines 284-291): The authors attribute the slight increase in HRFB from 2017-18 (57%) to 2022 (60%) to the COVID-19 pandemic. Soften this claim or present it as a hypothesis requiring further investigation. That is, the causal claim is speculative without:

• Supporting evidence or citations demonstrating pandemic impacts on HRFB specifically.

• Consideration of other potential explanations (e.g., sampling variation, changing demographics).

Comment 4—Religious affiliation interpretation (Lines 331-343): The finding that Muslim women have higher odds of multiple HRFB is presented with potentially overgeneralized explanations about "misconceptions about Islamic teachings" and "cultural norms." This requires:

• More nuanced discussion acknowledging diversity within religious groups.

• Consideration of confounding factors not fully captured in models.

• Careful language to avoid stereotyping or oversimplification.

• Revision to emphasize that religious affiliation may be a proxy for other unmeasured factors (e.g., community norms, healthcare access) rather than religion itself being causative.

Comment 5—In Table 2, some CIs are asymmetrical (e.g., “16.45(16.49,17.36)” appears to have an error as the lower bound exceeds the point estimate). Standardize reporting to 2 decimal places throughout and verify all intervals.

Comment 6—Some p-values are reported as “p<0.01” while others use asterisks. Consider using a consistent system (either all p-values or all asterisks).

Comment 7—Multilevel model specification: The authors do not clearly specify:

• Whether random intercepts or random slopes were used.

• The levels of clustering (household? community? district?)

• Justification of the multilevel approach.

• The authors did not use interaction terms in their models, and this is a limitation that should be addressed.

• The manuscript mentions only the Variance Inflation Factor (VIF) for multicollinearity assessment, but is completely silent on other essential model diagnostics.

Comment 8—Missing data handling: The manuscript does not describe how missing data were handled (DOI: 10.19139/soic-2310-5070-2894). Given the large sample, even small percentages missing could affect results.

Comment 9—Include a brief subsection in the Discussion explicitly linking findings to current Bangladesh health policies.

Comment 10—Reproducibility: Provide the exact code used for data cleaning, variable creation (e.g., the HRFB categories), weighting, and all models (multilevel logistic and multinomial, concentration indices). The authors should add a line in the Data Availability, Methods, or Supporting Information section of the manuscript: “The Stata do-file used for the data analysis is available as Supporting Information (S1 File).”

Comment 11—The authors need to demonstrate that their use of the chi-squared test was appropriate, given their data structure, and that the key assumptions were met.

Comment 12—The totals in Table 1 are inconsistent across variables, which suggests either:

• Missing data not properly accounted for.

• Reporting errors.

• Major subsample without explanation.

Recommendation: The authors must revise Table 1 to clearly indicate the base population for each variable and ensure all frequencies within each subsample are consistent. This is essential for transparency and reproducibility.

Comment 13—Using “1” in Table 4 to indicate reference categories could be misinterpreted. I strongly recommend changing all “1” entries to "Ref." and adding a clear footnote.

Comment 14—References are inconsistently formatted (some with DOIs, some without; some with truncated). PLOS ONE has specific reference formatting requirements that should be followed.

• In the text (Lines 171-172): The authors write: “The CIX was calculated using the 'convenient covariance' formula proposed by O'Donnell et al. [41].” In the reference list: [41] is Jann B. (2016). This is a miscitation. The authors must fix this citation error and re-verify all subsequent reference numbers.

• [27] and [56] are the same.

• [29] and [33] are the same.

• [31] and [62] are the same.

• There is no reference [63] in the list.

• The authors should carefully review their entire reference list and all in-text citations to ensure consistency and accuracy.

Comment 15—The paper contains enough typos and grammatical errors to require a thorough language revision.

Reviewer #3: Summary

The manuscript addresses an important public health issue using nationally representative data from the Bangladesh Demographic and Health Survey. The analysis is generally appropriate, and the findings are potentially valuable. However, a few aspects of clarity and methodological description require minor revision.

Minor Comments:

Clarity of Methods in Abstract:

The abstract could be improved for clarity by presenting the methods more clearly. In particular, the definition of high-risk fertility behaviour (HRFB) and the description of the outcome variables are somewhat difficult to follow. Consider revising to achieve a more concise, structured presentation.

Outcome Variables:

The manuscript states that both binary and categorical HRFB variables were constructed, but the rationale is not given. Please briefly justify this choice (e.g., to capture both the occurrence and the intensity of risk) and clearly describe the categorical specification.

Wording Issue:

The phrase ``designed on data from'' is awkward and should be revised (e.g., ``based on data from'' or ``using data from'').

Socioeconomic Disparities:

The sentence describing the assessment of socioeconomic disparities could be streamlined for clarity. Consider revising to: ``Socioeconomic inequalities in HRFB were assessed using concentration curves and concentration indices (CIX).''

Survey Design Specification and Models Goodness-of-fit:

The manuscript would benefit from clearer reporting of the statistical methodology. Specifically, stating that sampling weights were applied is not sufficient for a valid analysis of data from the {Bangladesh Demographic and Health Survey}. Please clarify whether clustering (PSU) and stratification were also accounted for in the analysis. In addition, no model fit statistics or diagnostic checks (e.g., ICC, AIC/BIC, multicollinearity) are reported. Including these would strengthen the validity and transparency of the analysis.

Wald Confidence Intervals vs. p-values:

Some Wald 95\% confidence intervals do not include the null value (0) even though the corresponding p-values are greater than 0.05. Please check the calculation method for the CIs and p-values and clarify which approach was used, particularly for the multilevel logistic and multinomial regression models.

Clarification of Models in Table 2:

Table 2 shows three columns (Any HRFB, Single HRFB, Multiple HRFB), which could be misinterpreted as three separate models. Please clarify whether a single multinomial logistic regression was fitted for the categorical HRFB outcome, with coefficients reported for each comparison, or whether separate models were used.

**********

what does this mean?). If published, this will include your full peer review and any attached files.

If you choose “no”, your identity will remain anonymous but your review may still be made public.

**Do you want your identity to be public for this peer review?**  For information about this choice, including consent withdrawal, please see our Privacy Policy

Reviewer #1: No

Reviewer #2: **Yes:**  Mahmoud A. Abdel-Fattah

Reviewer #3: No

**********

[NOTE: If reviewer comments were submitted as an attachment file, they will be attached to this email and accessible via the submission site. Please log into your account, locate the manuscript record, and check for the action link "View Attachments". If this link does not appear, there are no attachment files.]

To ensure your figures meet our technical requirements, please review our figure guidelines: https://journals.plos.org/plosone/s/figures

You may also use PLOS’s free figure tool, NAAS, to help you prepare publication quality figures: https://journals.plos.org/plosone/s/figures#loc-tools-for-figure-preparation.

NAAS will assess whether your figures meet our technical requirements by comparing each figure against our figure specifications.

Attachment Submitted filename: PONE-D-25-25913_comments.pdf

# Author comment: Author response to Decision Letter 1

**DOI:** 10.1371/journal.pone.0356299.r002

16 Apr 2026

Journal Requirements:

Requirement 1: Please ensure that your manuscript meets PLOS ONE's style requirements, including those for file naming. The PLOS ONE style templates can be found at

https://journals.plos.org/plosone/s/file?id=wjVg/PLOSOne_formatting_sample_main_body.pdf and

https://journals.plos.org/plosone/s/file?id=ba62/PLOSOne_formatting_sample_title_authors_affiliations.pdf

Response: Thanks for the comment. We reviewed the PLOS ONE style requirements and update the manuscript and file names accordingly, following the provided templates.

Requirement 2: Please provide additional details regarding participant consent. In the ethics statement in the Methods and online submission information, please ensure that you have specified (1) whether consent was informed and (2) what type you obtained (for instance, written or verbal, and if verbal, how it was documented and witnessed). If your study included minors, state whether you obtained consent from parents or guardians. If the need for consent was waived by the ethics committee, please include this information.

If you are reporting a retrospective study of medical records or archived samples, please ensure that you have discussed whether all data were fully anonymized before you accessed them and/or whether the IRB or ethics committee waived the requirement for informed consent. If patients provided informed written consent to have data from their medical records used in research, please include this information.

Response: Thank you for this helpful comment. We have revised the Ethics section to clarify that the 2022 BDHS obtained ethical approval from the ICF Institutional Review Board and the Bangladesh Medical Research Council (BMRC), and that informed, voluntary consent was obtained from all participants, with parental/guardian consent for minors. We also state that the publicly available BDHS datasets are fully anonymized, so no additional approval or consent was required for this secondary analysis. These details are now included in the revised manuscript

Requirement 3: In the online submission form, you indicated that data is publicly available on request on the DHS websites (https://www.dhsprogram.com/data/available-datasets.cfm).

All PLOS journals now require all data underlying the findings described in their manuscript to be freely available to other researchers, either a. In a public repository, b. Within the manuscript itself, or c. Uploaded as supplementary information.

This policy applies to all data except where public deposition would breach compliance with the protocol approved by your research ethics board. If your data cannot be made publicly available for ethical or legal reasons (e.g., public availability would compromise patient privacy), please explain your reasons on resubmission and your exemption request will be escalated for approval.

Response: Thank you for this clarification. In addition to noting that the original BDHS data are accessible upon registration through the DHS Program, we have now provided the dataset underlying our analyses as an Excel file in the Supporting Information (S1 File). This ensures that all data used in the manuscript are freely available to other researchers in accordance with the PLOS data availability policy.

Requirement 4: When completing the data availability statement of the submission form, you indicated that you will make your data available on acceptance. We strongly recommend all authors decide on a data sharing plan before acceptance, as the process can be lengthy and hold up publication timelines. Please note that, though access restrictions are acceptable now, your entire data will need to be made freely accessible if your manuscript is accepted for publication. This policy applies to all data except where public deposition would breach compliance with the protocol approved by your research ethics board. If you are unable to adhere to our open data policy, please kindly revise your statement to explain your reasoning and we will seek the editor's input on an exemption. Please be assured that, once you have provided your new statement, the assessment of your exemption will not hold up the peer review process.

Response: Thank you for this important clarification. The primary data used in this study are individual level microdata from the Bangladesh Demographic and Health Survey (BDHS) 2022, which are owned and managed by the DHS Program. Access to these datasets requires prior registration and approval from the DHS Program, and our data use agreement does not permit us to republish or publicly deposit the microdata (or any subset thereof) under an open licence.

In light of this, we are unable to make the full BDHS 2022 dataset freely available in a public repository. We have therefore revised our Data Availability statement to clarify that: (i) all BDHS 2022 datasets can be obtained directly from the DHS Program upon registration and approval (https://www.dhsprogram.com/data/available-datasets.cfm), and (ii) we provide the complete Stata do file used for data cleaning, variable construction, survey weighting, and all analyses as Supporting Information, so that any researcher who gains access to the BDHS 2022 data can fully reproduce our results.

Because restricting public redistribution of the DHS microdata is a condition of the original data use protocol and data ownership policy, we kindly request an exemption from the requirement to deposit the full analysis dataset in an open repository. We hope that the combination of controlled access via the DHS Program and fully shared analysis code will be acceptable under PLOS ONE’s data policy.

Requirement 5: Please ensure that you refer to Figure 2 in your text as, if accepted, production will need this reference to link the reader to the figure.

Response: Thank you for this reminder. In the revised manuscript, the former Figure 2 has been renumbered as Figure 3, and it is already explicitly cited in the main text to guide readers to the figure.

Requirement 6: Please remove your figures from within your manuscript file, leaving only the individual TIFF/EPS image files, uploaded separately. These will be automatically included in the reviewers’ PDF.

Response: Thank you for the instruction. All figures have been removed from the main manuscript file, and the individual figure files have been uploaded separately in TIFF format as requested.

Requirement 7: We note that Figure 2 in your submission contain map images which may be copyrighted. All PLOS content is published under the Creative Commons Attribution License (CC BY 4.0), which means that the manuscript, images, and Supporting Information files will be freely available online, and any third party is permitted to access, download, copy, distribute, and use these materials in any way, even commercially, with proper attribution. For these reasons, we cannot publish previously copyrighted maps or satellite images created using proprietary data, such as Google software (Google Maps, Street View, and Earth). For more information, see our copyright guidelines: http://journals.plos.org/plosone/s/licenses-and-copyright.

We require you to either (a) present written permission from the copyright holder to publish these figures specifically under the CC BY 4.0 license, or (b) remove the figures from your submission:

a. You may seek permission from the original copyright holder of Figure 2 to publish the content specifically under the CC BY 4.0 license.

We recommend that you contact the original copyright holder with the Content Permission Form (http://journals.plos.org/plosone/s/file?id=7c09/content-permission-form.pdf) and the following text:

“I request permission for the open-access journal PLOS ONE to publish XXX under the Creative Commons Attribution License (CCAL) CC BY 4.0 (http://creativecommons.org/licenses/by/4.0/). Please be aware that this license allows unrestricted use and distribution, even commercially, by third parties. Please reply and provide explicit written permission to publish XXX under a CC BY license and complete the attached form.”

Please upload the completed Content Permission Form or other proof of granted permissions as an ""Other"" file with your submission.

In the figure caption of the copyrighted figure, please include the following text: “Reprinted from [ref] under a CC BY license, with permission from [name of publisher], original copyright [original copyright year].”

b. If you are unable to obtain permission from the original copyright holder to publish these figures under the CC BY 4.0 license or if the copyright holder’s requirements are incompatible with the CC BY 4.0 license, please either i) remove the figure or ii) supply a replacement figure that complies with the CC BY 4.0 license. Please check copyright information on all replacement figures and update the figure caption with source information. If applicable, please specify in the figure caption text when a figure is similar but not identical to the original image and is therefore for illustrative purposes only.

The following resources for replacing copyrighted map figures may be helpful:

USGS National Map Viewer (public domain): http://viewer.nationalmap.gov/viewer/

The Gateway to Astronaut Photography of Earth (public domain): http://eol.jsc.nasa.gov/sseop/clickmap/

Maps at the CIA (public domain): https://www.cia.gov/library/publications/the-world-factbook/index.html and https://www.cia.gov/library/publications/cia-maps-publications/index.html

NASA Earth Observatory (public domain): http://earthobservatory.nasa.gov/

Landsat: http://landsat.visibleearth.nasa.gov/

USGS EROS (Earth Resources Observatory and Science (EROS) Center) (public domain): http://eros.usgs.gov/#

Natural Earth (public domain): http://www.naturalearthdata.com/

Response: Thank you for raising this issue. Thank you for your comment regarding Figure 3. We confirm that the map is not subject to copyright restrictions. The figure was created entirely by the authors using ArcGIS version 10.8 to illustrate the division‑wise distribution of HRFB across the different divisions of Bangladesh. The divisional shapefile used to prepare this map was obtained from the Humanitarian Data Exchange (HDX), an open‑source platform managed by the United Nations Office for the Coordination of Humanitarian Affairs (https://data.humdata.org/dataset/cod-ab-bgd), which is freely available for public use without licensing restrictions. As the map was generated from open‑source data and original analysis, it fully complies with the Creative Commons Attribution License (CC BY 4.0) requirements for publication in PLOS ONE.

Requirement 8: If the reviewer comments include a recommendation to cite specific previously published works, please review and evaluate these publications to determine whether they are relevant and should be cited. There is no requirement to cite these works unless the editor has indicated otherwise.

Response: Thank you for this clarification

Response Reviewer’s Comments

Reviewer #1:

Comment 1: Material and Methods: Please draw a flowchart to illustrate the conceptual framework.

Response: Thank you for this helpful suggestion. We agree that the earlier version did not sufficiently explain the rationale for selecting explanatory variables. We have now added a brief conceptual framework in the Methods section (Fig 1), outlining the pathways linking sociodemographic, household , and community-level factors to HRFB and citing the key theoretical and empirical literature that informed our covariate selection.

Comment 2: Figures: Map - Add a title

Response: Thank you for these helpful suggestion. We have also added an informative title to the map to improve its clarity and interpretability (Fig 3).

Reviewer #2:

Comment 1: Missing justification for variable selection: While the authors state they “reviewed the most recent relevant articles” (Line 130), they do not provide a theoretical framework or conceptual model guiding variable selection. Add a brief conceptual framework or cite specific theoretical models that informed covariate selection.

Response: Thank you for this helpful suggestion. We agree that the earlier version did not sufficiently explain the rationale for selecting explanatory variables. We have now added a brief conceptual framework in the Methods section (Fig 1), outlining the pathways linking sociodemographic, household , and community-level factors to HRFB and citing the key theoretical and empirical literature that informed our covariate selection.

Comment 2: Husband's age exclusion (Line 156): The authors excluded the husband's age due to high collinearity, but do not report:

• The VIF values that led to this decision.

• Whether sensitivity analyses were conducted with/without this variable.

• The first two categories for husband's age in Table 1 overlap at age 30. A husband who is exactly 30 years old could logically be placed in either category. This violates the statistical principle that categories should be mutually exclusive and collectively exhaustive. If these categories were used in the regression (Table 4), the categorization error would affect the results (though husband's age was excluded from the regression due to collinearity, the descriptive table is still incorrect).

• Potential implications of excluding an important demographic factor.

• Is excluding variables the best solution? Consider adding possible alternative solutions to the limitations (DOI: 10.1080/03610918.2019.1711409).

Response: Thank you for these detailed and helpful comments. We apologize for the confusion caused by the overlapping husband’s age categories in Tables 1 and 3. This was a typographical error: the first category should have been 15–29 years, and the second 30–34 years. We have corrected these categories so that they are now mutually exclusive and collectively exhaustive, and a husband who is exactly 30 years old is included in the 30–34 years group in the revised tables. Regarding collinearity, we have now reported the Variance Inflation Factor (VIF) that led to the exclusion of husband’s age from the multivariable models. The VIF for husband’s age was 6.72, which we considered indicative of problematic multicollinearity. We have added this information to the Statistical Analysis subsection of the Methods (line 168-173). We also conducted sensitivity analyses including and excluding husband’s age from the multivariable models. These analyses showed that the direction and magnitude of the associations between the main covariates and HRFB were materially unchanged when husband’s age was included. We agree that excluding an important demographic factor is not ideal. In the Limitations section (line 407-411), we have added a short paragraph noting that we opted to exclude husband’s age due to multicollinearity, and we acknowledge that alternative approaches to handling multicollinearity (as discussed in the referenced article) could be considered in future work.

Comment 3: Causal attribution to COVID-19 (Lines 284-291): The authors attribute the slight increase in HRFB from 2017-18 (57%) to 2022 (60%) to the COVID-19 pandemic. Soften this claim or present it as a hypothesis requiring further investigation. That is, the causal claim is speculative without:

• Supporting evidence or citations demonstrating pandemic impacts on HRFB specifically.

• Consideration of other potential explanations (e.g., sampling variation, changing demographics).

Response: We thank the reviewer for this important comment. We agree that our initial wording could be interpreted as implying a causal effect of the COVID 19 pandemic on the observed increase in HRFB. We have therefore revised the discussion to present the pandemic as a possible contributing factor rather than a definitive cause and to acknowledge that this interpretation is speculative and would require further investigation with appropriate data. We also note that other explanations, such as sampling variation and underlying demographic or service related changes, may have contributed to the observed difference. (line 310-317)

Comment 4: Religious affiliation interpretation (Lines 331-343): The finding that Muslim women have higher odds of multiple HRFB is presented with

Attachment Submitted filename: Response to Reviewers.pdf

# Peer review: Decision Letter 1

**DOI:** 10.1371/journal.pone.0356299.r003

**Authors:** Ahmed Jubayer

16 Jul 2026

Dear Dr. Noor,

Thank you for submitting your manuscript to PLOS ONE. After careful consideration, we feel that it has merit but does not fully meet PLOS ONE’s publication criteria as it currently stands. Therefore, we invite you to submit a revised version of the manuscript that addresses the points raised during the review process.

Please submit your revised manuscript by Aug 30 2026 11:59PM. If you will need more time than this to complete your revisions, please reply to this message or contact the journal office at plosone@plos.org. When you're ready to submit your revision, log on to https://www.editorialmanager.com/pone/ and select the 'Submissions Needing Revision' folder to locate your manuscript file.

- A letter that responds to each point raised by the academic editor and reviewer(s). You should upload this letter as a separate file labeled 'Response to Reviewers'.
- A marked-up copy of your manuscript that highlights changes made to the original version. You should upload this as a separate file labeled 'Revised Manuscript with Track Changes'.
- An unmarked version of your revised paper without tracked changes. You should upload this as a separate file labeled 'Manuscript'.

If you would like to make changes to your financial disclosure, please include your updated statement in your cover letter. Guidelines for resubmitting your figure files are available below the reviewer comments at the end of this letter.

If applicable, we recommend that you deposit your laboratory protocols in protocols.io to enhance the reproducibility of your results. Protocols.io assigns your protocol its own identifier (DOI) so that it can be cited independently in the future. For instructions see: https://journals.plos.org/plosone/s/submission-guidelines#loc-laboratory-protocols. Additionally, PLOS ONE offers an option for publishing peer-reviewed Lab Protocol articles, which describe protocols hosted on protocols.io. Read more information on sharing protocols at https://plos.org/protocols?utm_medium=editorial-email&utm_source=authorletters&utm_campaign=protocols.

As the corresponding author, your ORCID iD is verified in the submission system and will appear in the published article. PLOS supports the use of ORCID, and we encourage all coauthors to register for an ORCID iD and use it as well. Please encourage your coauthors to verify their ORCID iD within the submission system before final acceptance, as unverified ORCID iDs will not appear in the published article. *Only*  the individual author can complete the verification step; PLOS staff *cannot*  verify ORCID iDs on behalf of authors.

We look forward to receiving your revised manuscript.

Kind regards,

Ahmed Jubayer

Academic Editor

PLOS One

Journal Requirements:

If the reviewer comments include a recommendation to cite specific previously published works, please review and evaluate these publications to determine whether they are relevant and should be cited. There is no requirement to cite these works unless the editor has indicated otherwise.

[Note: HTML markup is below. Please do not edit.]

Reviewers' comments:

Reviewer's Responses to Questions

**Comments to the Author**

Reviewer #2: (No Response)

Reviewer #3: All comments have been addressed

**********

2. Is the manuscript technically sound, and do the data support the conclusions?

Reviewer #2: Partly

Reviewer #3: Yes

**********

3. Has the statistical analysis been performed appropriately and rigorously?

Reviewer #2: No

Reviewer #3: Yes

**********

4. Have the authors made all data underlying the findings in their manuscript fully available?

The PLOS Data policy

Reviewer #2: No

Reviewer #3: Yes

**********

5. Is the manuscript presented in an intelligible fashion and written in standard English?

Reviewer #2: Yes

Reviewer #3: Yes

**********

Reviewer #2: Journal: PLOS ONE

MS ID: PONE-D-25-25913R1

Title: Trends, patterns, determinants and socio-economic inequality of high-risk fertility behavior (HRFB) among Bangladeshi women: evidence from Bangladesh Demographic Health Survey

Version: R1 Date: 12 May 2026

Reviewer's report:

Below is my structured assessment of the remaining weaknesses and specific improvements still required.

Comment 1—In the revised submission, the competing interests statement reads: “R.B.I and S.T.A.N are husband and wife”. Does the authors' marital relationship constitute a competing interest? PLOS ONE typically requires disclosure of any financial or personal relationships that could bias the work. Being married is not usually considered a competing interest unless it influences authorship or peer review.

Comment 2—We read: “For transparency and reproducibility, we have provided the subset of the BDHS 2022 data that was used in our analyses as an anonymized Excel file in the Supporting Information (S1 File).” Indeed, the data file is not available.

Comment 3—The authors deleted approximately 9,550 women due to missing data. The authors never specify which variables had missing data or how much missingness each variable had. According to STROBE guidelines (which the authors claim to follow), they should report:

• The number of missing values for each variable (not just the final N).

• The reasons for missingness (if known).

• A comparison of included vs. excluded participants to assess potential bias.

The manuscript reports none of these.

Comment 4—Table 1: Add a footnote explaining the small discrepancies (i.e., summed N: Religion (19,055); Husband's age (19,058)) and the exclusion of ANC/delivery variables. While small discrepancies can occur due to weighting and rounding, the authors should either: Reconcile them to show a consistent N = 19,060 across all full-sample variables or add a footnote explaining that small variations are due to weighted rounding.

Comment 5—The authors found that the husband's age had a VIF of 6.72, which exceeded their threshold for problematic multicollinearity. The authors handled this reasonably, not perfectly. They responded by excluding the husband's age from the multivariable models entirely. Deleting the husband's age was an acceptable but suboptimal solution. Logistic ridge regression would have been better. For a revision at this stage, I would accept their approach provided they add the sensitivity analysis results to the manuscript (not just the response letter). Alternative approaches specifically designed for multicollinearity in binomial logistic regression, such as ridge-type estimators [DOI: 10.1080/03610918.2019.1711409], could be employed in future research to retain all predictors while stabilizing coefficient estimates.

Comment 6—Limitations:

• The authors have acknowledged that multilevel model diagnostics are needed, but they have not actually performed or reported them. This is insufficient. Without diagnostic checks, the model assumptions remain unverified.

• The limitations section in the revised manuscript is missing the fourth numbered item. The authors should renumber the limitations sequentially in the next revision. I strongly recommend making this correction now rather than waiting for the proof stage.

Comment 7—Reference list:

• [27] and [56] are duplicated (Identical reference appears twice as noted in the previous review). Remove [56] and renumber.

• [25] and [34] are duplicated (Same authors, same journal, same year, same DOI). Remove one and renumber.

• Inconsistent DOI formatting: e.g, (doi:10.1186/... for []6, while doi:https://doi.org/... for [37], [38]). Choose one format and apply it to all.

Reviewer #3: The authors have adequately addressed the concerns raised in the previous review rounds. The manuscript has improved substantially in clarity, presentation, and methodological explanation. In its current form, the paper is suitable for publication.

**********

what does this mean?). If published, this will include your full peer review and any attached files.

If you choose “no”, your identity will remain anonymous but your review may still be made public.

**Do you want your identity to be public for this peer review?**  For information about this choice, including consent withdrawal, please see our Privacy Policy

Reviewer #2: **Yes:**  Mahmoud A. Abdel-Fattah

Reviewer #3: No

**********

[NOTE: If reviewer comments were submitted as an attachment file, they will be attached to this email and accessible via the submission site. Please log into your account, locate the manuscript record, and check for the action link "View Attachments". If this link does not appear, there are no attachment files.]

To ensure your figures meet our technical requirements, please review our figure guidelines: https://journals.plos.org/plosone/s/figures

You may also use PLOS’s free figure tool, NAAS, to help you prepare publication quality figures: https://journals.plos.org/plosone/s/figures#loc-tools-for-figure-preparation.

NAAS will assess whether your figures meet our technical requirements by comparing each figure against our figure specifications.

# Author comment: Author response to Decision Letter 2

**DOI:** 10.1371/journal.pone.0356299.r004

1 Aug 2026

Response to reviewer:

Comment 1: In the revised submission, the competing interests statement reads: "R.B.I and S.T.A.N are husband and wife". Does the authors' marital relationship constitute a competing interest? PLOS ONE typically requires disclosure of any financial or personal relationships that could bias the work. Being married is not usually considered a competing interest unless it influences authorship or peer review.

Response: We thank the reviewer for this clarification. We agree that a marital relationship between co-authors does not, by itself, constitute a competing interest under PLOS ONE's policy, as it did not influence the assignment of authorship, the peer review process, or the interpretation of the findings. We had included this statement solely in the interest of full transparency regarding author relationships, rather than as a declaration of bias. We have revised the Competing Interests statement to remove this line and have instead confirmed that "The authors have declared that no competing interests exist," consistent with PLOS ONE's guidance.

Comment 2: We read: "For transparency and reproducibility, we have provided the subset of the BDHS 2022 data that was used in our analyses as an anonymized Excel file in the Supporting Information (S1 File)." Indeed, the data file is not available.

Response: We thank the reviewer for flagging this. We initially intended to share a subset of the BDHS 2022 data as an anonymized Excel file for transparency. However, upon further review of the DHS Program's data-use terms, we found that redistributing any subset of the BDHS microdata, even in anonymized form, is not permitted without explicit authorization from the DHS Program, as access is governed by a registration and data-use agreement that restricts public redistribution. In light of this, we removed the Excel data file from the Supporting Information and instead updated our Data Availability statement to reflect that the BDHS 2022 dataset can be obtained directly from the DHS Program upon registration and approval, and that we have provided the complete Stata do-file used for data cleaning, variable construction, survey weighting, and all analyses as Supporting Information, so that any researcher who obtains the BDHS 2022 data can fully reproduce our results. We apologize that this revision may not have been clearly flagged in our previous response letter, and we confirm that the updated Data Availability statement and the Stata do-file are both included in the current resubmission.

Comment 3: The authors deleted approximately 9,550 women due to missing data. The authors never specify which variables had missing data or how much missingness each variable had. According to STROBE guidelines (which the authors claim to follow), they should report: • The number of missing values for each variable (not just the final N). • The reasons for missingness (if known). • A comparison of included vs. excluded participants to assess potential bias. The manuscript reports none of these.

Response: We thank the reviewer for this important point and apologise for not making the reason for this exclusion clear in the original submission. The great majority of the 9,550 excluded women were not missing data at random; rather, their missingness reflects the BDHS 2022 questionnaire design itself. As described in the survey's own methodology (Section 1.9, Response Rates), only a subsample of eligible ever-married women (20,217 of 30,358) were administered the full questionnaire, which alone collects husband/partner characteristics (e.g., husband's age, husband's education, husband's occupation) and age at first marriage/cohabitation. The remaining eligible women (10,141) received the short questionnaire, which does not include these modules. Because several of our key covariates, specifically husband's age, husband's education, and age at marriage, are only available for women who received the full questionnaire, women who were administered the short questionnaire are, by design, missing on these variables and were therefore excluded from our analytic sample. This is a structural feature of the survey's sub-sampling design rather than item-level non-response, and we have revised the manuscript to state this explicitly

Comment 4: Table 1: Add a footnote explaining the small discrepancies (i.e., summed N: Religion (19,055); Husband's age (19,058)) and the exclusion of ANC/delivery variables. While small discrepancies can occur due to weighting and rounding, the authors should either: reconcile them to show a consistent N = 19,060 across all full-sample variables or add a footnote explaining that small variations are due to weighted rounding.

Response: We thank the reviewer for this observation. We reviewed the underlying data and confirmed that the small discrepancies in summed N across variables (e.g., Religion: 19,055; Husband's age: 19,058, against the full analytic sample of N=19,060) arise from a combination of weighted rounding and a small number of missing/don't-know responses for specific variables, rather than any inconsistency in the underlying analytic sample. As reconciling every variable to an identical summed N would require either dropping cases with any missing values on any variable or artificially imputing missing values, both of which we consider undesirable for a descriptive table, we have instead added an explanatory footnote to Table 1 clarifying this for the reader.

Comment 5: The authors found that the husband's age had a VIF of 6.72, which exceeded their threshold for problematic multicollinearity. The authors handled this reasonably, not perfectly. They responded by excluding the husband's age from the multivariable models entirely. Deleting the husband's age was an acceptable but suboptimal solution. Logistic ridge regression would have been better. For a revision at this stage, I would accept their approach provided they add the sensitivity analysis results to the manuscript (not just the response letter). Alternative approaches specifically designed for multicollinearity in binomial logistic regression, such as ridge-type estimators [DOI: 10.1080/03610918.2019.1711409], could be employed in future research to retain all predictors while stabilizing coefficient estimates.

Response: We thank the reviewer for this guidance and confirm that we have now included the full sensitivity analysis results directly in the manuscript as Supplementary Table S1, rather than presenting them only in this response letter. Supplementary Table S1 presents our primary multivariable multilevel model alongside a sensitivity model that additionally includes the husband's age, allowing direct comparison of adjusted odds ratios and 95% confidence intervals across both specifications.

This comparison shows that all covariates in our primary model, respondent's and husband's education, household size, media exposure, wealth index, religion, contraceptive use, age at marriage, place of residence, and administrative division, remain materially unchanged in both magnitude and direction when husband's age is included.

Comment 6: Limitations: • The authors have acknowledged that multilevel model diagnostics are needed, but they have not actually performed or reported them. This is insufficient. Without diagnostic checks, the model assumptions remain unverified. • The limitations section in the revised manuscript is missing the fourth numbered item. The authors should renumber the limitations sequentially in the next revision. I strongly recommend making this correction now rather than waiting for the proof stage.

Response: We thank the reviewer for catching both issues. Regarding the numbering, we confirm this was an oversight (the list currently jumps from "Third" to "Fifth" with no "Fourth" item) and we have corrected the sequential numbering throughout. Regarding model diagnostics, we agree that stating diagnostics "are needed" without performing them is insufficient. We have now conducted and report basic multilevel model diagnostics, including the intraclass correlation coefficient (ICC) to confirm meaningful cluster-level variance justifying the multilevel approach, and a check of random-effects normality. We have revised the corresponding limitation point to accurately reflect what was and was not diagnostically verified, rather than deferring this to future work.

Comment 7: Reference list: • [27] and [56] are duplicated (Identical reference appears twice as noted in the previous review). Remove [56] and renumber. • [25] and [34] are duplicated (Same authors, same journal, same year, same DOI). Remove one and renumber. • Inconsistent DOI formatting: e.g., (doi:10.1186/... for [16], while doi:https://doi.org/... for [37], [38]). Choose one format and apply it to all.

Response: We thank the reviewer for identifying these duplicate entries. We have removed reference [56] (retaining [27]) and reference [34] (retaining [25]) as instructed, and renumbered the reference list and all corresponding in-text citations sequentially throughout the manuscript. In auditing the full reference list to address this comment, we identified three additional duplicate pairs that had not previously been flagged: Amir-ud-Din et al. (originally [6] and [52]), Pal & Shekhar (originally [7] and [32]), and the Wagstaff et al. / O'Donnell et al. World Bank health equity guide (originally [39] and [40], which shared an identical DOI despite differing author-order formatting). We have removed the duplicate entry in each of these three additional pairs and renumbered accordingly, so the corrected reference list now contains 54 unique entries rather than 61. We have also standardised all DOI formatting to the bare "doi:10.xxxx" convention (removing the "https://doi.org/" prefix used inconsistently for references [37] and [38], and removing the inconsistent space after "doi:" that appeared in several other entries) so that DOI formatting is now uniform throughout the reference list.

# Peer review: Decision Letter 2

**DOI:** 10.1371/journal.pone.0356299.r005

**Authors:** Ahmed Jubayer

19 Aug 2026

Trends, patterns, determinants and socio-economic inequality of high-risk fertility behavior (HRFB) among Bangladeshi women: evidence from Bangladesh Demographic Health Survey

PONE-D-25-25913R2

Dear Dr. Noor,

We’re pleased to inform you that your manuscript has been judged scientifically suitable for publication and will be formally accepted for publication once it meets all outstanding technical requirements.

Within one week, you’ll receive an e-mail detailing the required amendments. When these have been addressed, you’ll receive a formal acceptance letter and your manuscript will be scheduled for publication.

An invoice will be generated when your article is formally accepted. Please note, if your institution has a publishing partnership with PLOS and your article meets the relevant criteria, all or part of your publication costs will be covered. Please make sure your user information is up-to-date by logging into Editorial Manager at Editorial Manager® and clicking the ‘Update My Information' link at the top of the page. For questions related to billing, please contact billing support.

If your institution or institutions have a press office, please notify them about your upcoming paper to help maximize its impact. If they’ll be preparing press materials, please inform our press team as soon as possible -- no later than 48 hours after receiving the formal acceptance. Your manuscript will remain under strict press embargo until 2 pm Eastern Time on the date of publication. For more information, please contact onepress@plos.org.

Kind regards,

Ahmed Jubayer

Academic Editor

PLOS One

Additional Editor Comments (optional):

Reviewers' comments:

Reviewer's Responses to Questions

**Comments to the Author**

Reviewer #2: All comments have been addressed

**********

2. Is the manuscript technically sound, and do the data support the conclusions?

Reviewer #2: Yes

**********

3. Has the statistical analysis been performed appropriately and rigorously?

Reviewer #2: Yes

**********

4. Have the authors made all data underlying the findings in their manuscript fully available?

The PLOS Data policy

Reviewer #2: No

**********

5. Is the manuscript presented in an intelligible fashion and written in standard English?

Reviewer #2: Yes

**********

Reviewer #2: Journal: PLOS ONE

MS ID: PONE-D-25-25913R2

Title: Trends, patterns, determinants and socio-economic inequality of high-risk fertility behavior (HRFB) among Bangladeshi women: evidence from Bangladesh Demographic Health Survey

Version: R2 Date: 19 August 2026

Reviewer's report:

I am pleased to recommend acceptance of this manuscript. The following remaining issues are minor. They do not invalidate the study's findings, but they reflect a lack of attention to detail in the manuscript preparation. An additional round of review is not necessary. The editorial office can verify these fixes during production.

Comment 1—Throughout the manuscript, there is inconsistent use of UK and US English (e.g., behaviour and behavior in the title of the paper). Choose one variant and apply it consistently.

Comment 2—The authors explicitly state in the Methods (Line 180-181): “Complete case analysis was performed, and participants with missing data on any of the study variables were excluded from the analytical model.”. If complete-case analysis was used, there should be no missing data in the analytic sample (N=19,060). The presence of a “Missing” row (n=5) in Table 1 directly contradicts this statement.

**********

what does this mean?). If published, this will include your full peer review and any attached files.

If you choose “no”, your identity will remain anonymous but your review may still be made public.

**Do you want your identity to be public for this peer review?**  For information about this choice, including consent withdrawal, please see our Privacy Policy

Reviewer #2: **Yes:**  Mahmoud A. Abdel-Fattah

**********

# Editor report: Acceptance letter

**DOI:** 10.1371/journal.pone.0356299.r006

**Authors:** Ahmed Jubayer

PONE-D-25-25913R2

PLOS One

Dear Dr. Noor,

I'm pleased to inform you that your manuscript has been deemed suitable for publication in PLOS One. Congratulations! Your manuscript is now being handed over to our production team.

At this stage, our production department will prepare your paper for publication. This includes ensuring the following:

* All references, tables, and figures are properly cited

* All relevant supporting information is included in the manuscript submission,

* There are no issues that prevent the paper from being properly typeset

You will receive further instructions from the production team, including instructions on how to review your proof when it is ready. Please keep in mind that we are working through a large volume of accepted articles, so please give us a few days to review your paper and let you know the next and final steps.

Lastly, if your institution or institutions have a press office, please let them know about your upcoming paper now to help maximize its impact. If they'll be preparing press materials, please inform our press team within the next 48 hours. Your manuscript will remain under strict press embargo until 2 pm Eastern Time on the date of publication. For more information, please contact onepress@plos.org.

You will receive an invoice from PLOS for your publication fee after your manuscript has reached the completed accept phase. If you receive an email requesting payment before acceptance or for any other service, this may be a phishing scheme. Learn how to identify phishing emails and protect your accounts at https://explore.plos.org/phishing.

If we can help with anything else, please email us at customercare@plos.org.

Thank you for submitting your work to PLOS One and supporting open access.

Kind regards,

PLOS One Editorial Office Staff

on behalf of

Mr. Ahmed Jubayer

Academic Editor

PLOS One
